# Inhibiting endothelial cell Mst1 attenuates acute lung injury in mice

**DOI:** 10.1172/jci.insight.178208

**Published:** 2024-09-10

**Authors:** Zhi-Fu Guo, Nopprarat Tongmuang, Chao Li, Chen Zhang, Louis Hu, Daniel Capreri, Mei-Xing Zuo, Ross Summer, Jianxin Sun

**Affiliations:** 1 Center for Translational Medicine, Thomas Jefferson University, Philadelphia, Pennsylvania, USA.; 2 Department of Cardiology, Changhai Hospital, Naval Medical University, Shanghai.

**Keywords:** Inflammation, Vascular biology, Cytokines, Endothelial cells, NF-kappaB

## Abstract

Lung endothelium plays a pivotal role in the orchestration of inflammatory responses to acute pulmonary insults. Mammalian sterile 20-like kinase 1 (Mst1) is a serine/threonine kinase that has been shown to play an important role in the regulation of apoptosis, stress responses, and organ growth. This study investigated the role of Mst1 in lung endothelial activation and acute lung injury (ALI). We found that Mst1 was significantly activated in inflamed lung endothelial cells (ECs) and mouse lung tissues. Overexpression of Mst1 promoted nuclear factor κ-B (NF-κB) activation through promoting JNK and p38 activation in lung ECs. Inhibition of Mst1 by either its dominant negative form (DN-Mst1) or its pharmacological inhibitor markedly attenuated cytokine-induced expression of cytokines, chemokines, and adhesion molecules in lung ECs. Importantly, in a mouse model of lipopolysaccharide-induced (LPS-induced) ALI, both deletion of Mst1 in lung endothelium and treatment of WT mice with a pharmacological Mst1 inhibitor significantly protected mice from LPS-induced ALI. Together, our findings identified Mst1 kinase as a key regulator in controlling lung EC activation and suggest that therapeutic strategies aimed at inhibiting Mst1 activation might be effective in the prevention and treatment of inflammatory lung diseases.

## Abstract 2

Mst1 is activated in inflammatory lung endothelial cells and inhibition of Mst1 prevents development of acute lung injury in mice.

## Introduction

Acute lung injury (ALI) and its more severe form, acute respiratory distress syndrome (ARDS), are inflammatory lung disorders that cause significant morbidity and mortality in a considerable proportion of patients under hospitalization (1). The lung endothelium has been identified as an essential player in orchestrating and propagating ARDS (2), and disruption of lung endothelium is one of the major hallmarks of ARDS pathobiology (3, 4). Under normal physiological conditions, the lung endothelium exhibits an inhibitory effect on inflammation (5, 6). However, in response to a wide range of pathological insults, such as hypoxia, bacterial toxins, and stimulation of proinflammatory cytokines and chemokines, activation of the lung endothelium leads to the recruitment of inflammatory cells to vascular walls and the transmigration of leukocytes into the lung (7). As such, therapeutic strategies aimed at reducing endothelial activation are now believed to be important for decreasing the onset and severity of ARDS and other inflammatory vascular conditions (8, 9).

Activation of the transcription factor nuclear factor κ-B (NF-κB) plays an important role in mediating expression of adhesion molecules and the subsequent interaction of immune cells with endothelial cells (ECs) (10, 11). The promoter regions of many of the genes for adhesion molecules and proinflammatory cytokines normally have binding sites for NF-κB. In resting ECs, NF-κB resides as an inactive molecule in the cytoplasm by forming complexes with inhibitors, such as the IκB proteins (IκBs). However, upon stimulation by proinflammatory cytokines, IκBs are phosphorylated by IκB kinase (IKK), ubiquitinated, and proteolytically degraded by 26S proteasomes, which allows NF-κB to translocate to the nucleus, where it can bind to promoter regions and induce the transcription of key inflammatory target genes, such as a host of adhesion molecules, inflammatory cytokines, and chemokines (11). Notably, several therapeutic approaches to suppress inflammation are based on the ability to control NF-κB activation, although these have yet to prove effective in patients (4, 12). Therefore, further understanding the mechanisms underlying NF-κB activation will help for developing new therapies for inflammatory vascular diseases such as ALI and ARDS.

Mammalian sterile 20-like kinase 1 (Mst1), a mammalian homolog of Hippo, is a ubiquitously expressed serine/threonine kinase that has been implicated in regulating various cellular processes, including cell proliferation, apoptosis, and immune responses (13, 14). Indeed, in response to various stimuli and cellular stresses, including TNF-α, serum starvation, staurosporine, UV irradiation, and anticancer drugs, Mst1 is activated in a number of cell lines, including BJAB, 293T, COS-1, Jurkat, HeLa, and HL-60 (14). Further, Mst1 has been shown to be activated in response to ischemia/reperfusion in cardiac myocytes and other types of vascular injury (15–18), further suggesting its important roles in the pathogenesis of cardiovascular diseases. Although Mst1 is highly expressed in the lung (19, 20), its involvement in regulating signaling pathways of lung injury remains largely unknown. Investigating how Mst1 influences inflammatory responses in the lungs could provide valuable insights into the underlying mechanisms and potential therapeutic strategies for this condition.

In this study, we hypothesized that Mst1 might be involved in regulating endothelial inflammatory responses to pulmonary insults. To test this hypothesis, we employed both genetic and pharmacological approaches to systematically dissect the role of Mst1 in lung endothelial biology. In brief, our findings indicate that Mst1 is a critical regulator of inflammatory response in lung endothelium. More specifically, we show that Mst1 activation promotes endothelial inflammation and worsens lung injury to LPS whereas targeted deletion of Mst1 in lung endothelium or pharmacological inhibitors of Mst1/2 effectively mitigate LPS-induced inflammatory responses in the lung.

## Results

### Mst1 is activated in lung ECs after exposure to TNF-α.

**Figure 1:**
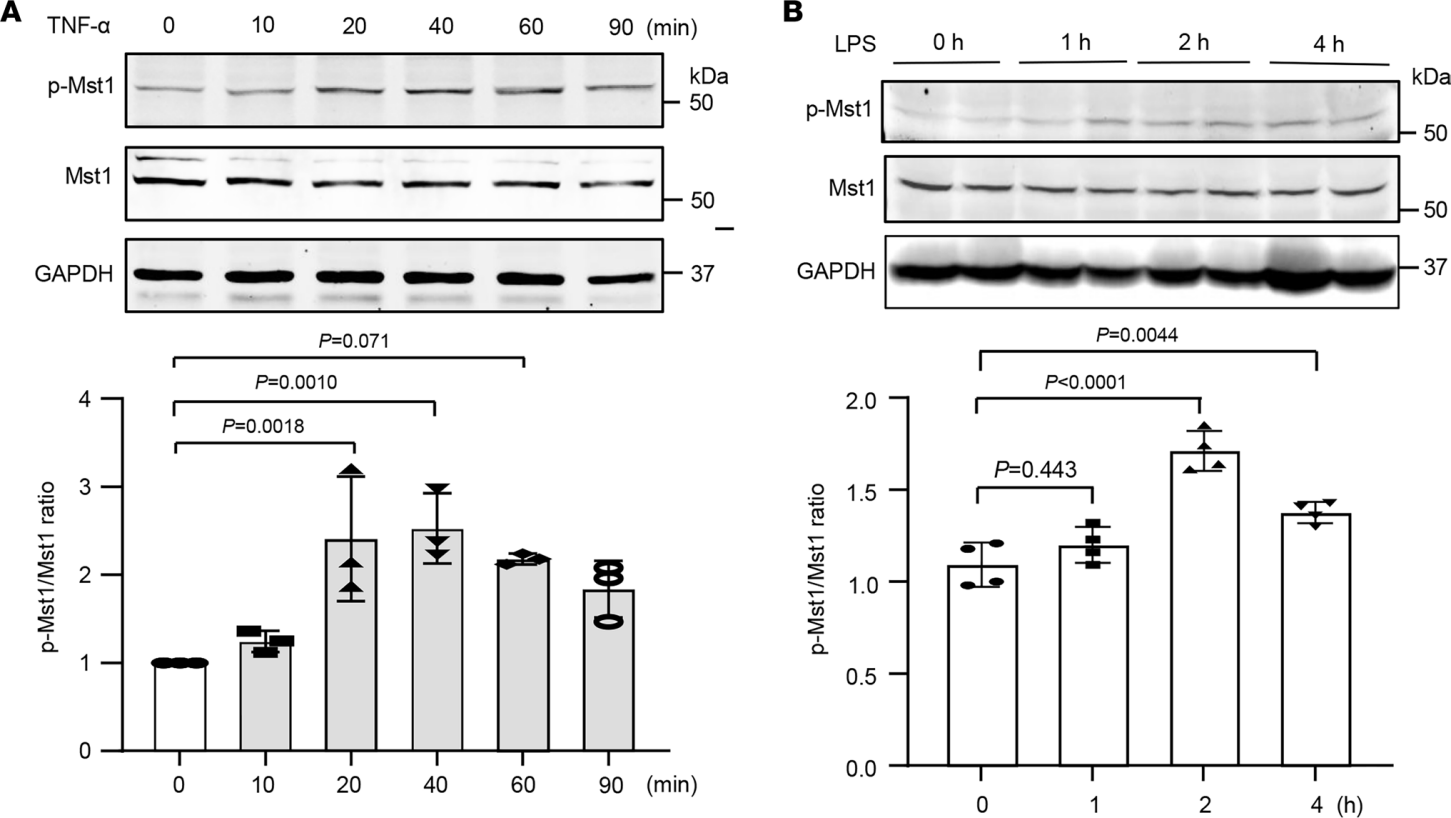
TNF-α induces Mst1 activation in lung ECs. (**A**) Lung ECs were treated with 20 ng/mL TNF-α at the indicated time points. Phosphorylation and total protein levels were determined by Western blot and results were quantified by densitometry analysis. *n* = 3. Statistical significance was determined by 1-way ANOVA with Šidák’s multiple comparisons. (**B**) Mice were subjected to LPS instillation. Lung tissues were collected for the determination of Mst1 phosphorylation by Western blot. *n* = 4. Statistical significance was determined by a 1-way ANOVA with Šidák’s multiple comparisons (**A** and **B**).

To investigate the role of Mst1 in pulmonary vascular inflammation, we examined the activation and expression of Mst1 in lung ECs in response to TNF-α, which is a recognized inducer of endothelial activation and plays an important role in the pathobiology of ALI and ARDS in humans (21, 22). The activation of Mst1, as indicated by its phosphorylation at Thr183 (23), was determined by Western blot at different time points after TNF-α stimulation. As shown in Figure 1A, TNF-α stimulation significantly increased Mst1 phosphorylation at Thr183 in a time-dependent manner. The maximal induction of TNF-α on Mst1 phosphorylation was observed at 20 minutes, when Mst1 phosphorylation was increased by approximately 2.5-fold. To determine whether Mst1 activation is involved in inflammatory lung diseases, we generated a mouse model of ALI induced by lipopolysaccharide (LPS) instillation (24). As shown in Figure 1B, Mst1 phosphorylation, as determined by Western blot, was significantly increased in a time-dependent manner in mouse lung tissues after LPS instillation. Further, we found that the increased levels of p-Mst1 were blunted in response to the second dose of LPS (Supplemental Figure 1; supplemental material available online with this article; https://doi.org/10.1172/jci.insight.178208DS1), suggesting that failure to upregulate p-Mst1 may contribute to LPS tolerance. Together, these data suggest that Mst1 may regulate inflammatory responses in lung ECs.

### Mst1 induces NF-κB activation.

**Figure 2:**
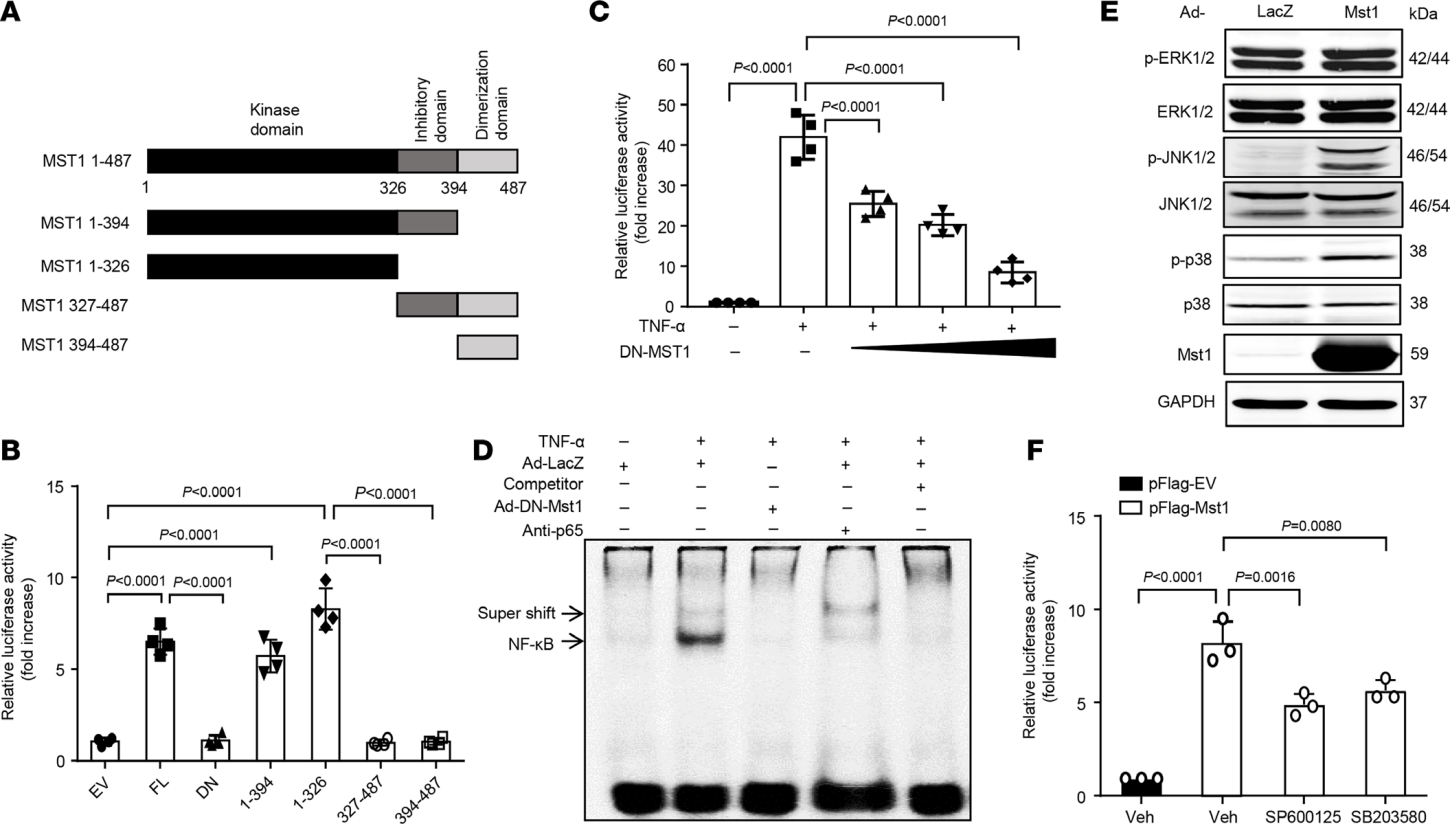
Mst1 induces NF-κB activation in ECs. (**A**) Schematic representation of Mst1 mutants. (**B**) EA.hy926 cells were transfected with 100 ng of p(NF-κB)3-Luc, 10 ng of pRL-RSV, and 200 ng of either empty vector (EV) or Mst1 mutants. Thirty-six hours after transfection, luciferase assays were performed (*n* = 4). (**C**) Lung ECs were transfected with 100 ng of p(NF-κB)3-Luc, 10 ng of pRL-RSV, and increasing amounts of pFlag-DN-Mst1. Thirty-six hours after transfection, luciferase assays were performed 6 hours after treatment with or without 20 ng/mL TNF-α (*n* = 4). (**D**) Lung ECs were transduced with adenoviruses bearing LacZ (Ad-lacZ) or DN-Mst1 (Ad-DN-Mst1) (at a multiplicity of infection [MOI] of 50) for 48 hours and then treated in the presence or absence of 20 ng/mL TNF-α for 1 hour. Nuclear protein was extracted, and EMSA was performed. The NF-κB complex was partially supershifted by anti–NF-κB p65 antibody or blocked by cold competitive probe. (**E**) Human lung ECs were transduced with Ad-LacZ or Ad-Mst1 (MOI, 50). Forty-eight hours after transduction, cell lysates were collected for Western blot analysis. (**F**) EA.hy926 cells were pretreated with DMSO vehicle control, JNK inhibitor (SP600125, 20 μmol/L) or p38 MAPK inhibitor (SB203580, 20 μmol/L) for 1 hour. Subsequently, the cells were transfected with 500 ng of p(NF-kB)3-Luc, 50 ng of pRL-RSV, 200 ng of either empty vector (pFlag-EV) or pFlag-Mst1. Twenty-four hours after transfection, luciferase assays were performed using Dual-Luciferase Reporter Assay System. Significance was determined by a 2-way ANOVA with Bonferroni’s post hoc test (**B**, **C**, and **F**).

Activation of the NF-κB signaling is essential for driving many inflammatory and immunological responses (10). To determine whether Mst1 plays a role in the regulation of pulmonary vascular inflammation, we examined the effect of Mst1 overexpression on NF-κB promoter activity at the transcriptional level in ECs. Mst1 contains an N-terminal kinase domain (aa 1–325), inhibitory domain (aa 326–394), and a C-terminal dimerization domain (aa 395–487) (25, 26) (Figure 2A). We generated a series of Mst1 deletion mutants and transfected these mutants into the EC line EA.hy296 cells along with a reporter plasmid containing a heterologous promoter driven by NF-κB elements upstream of the luciferase gene. As shown in Figure 2B, transfection of the full-length and Mst1 mutants containing its kinase domain significantly augmented the NF-κB promoter activity, whereas transfection of Mst1 dominant-negative (DN) mutant (K59R, DN-Mst1) (25), C-terminal inhibitory domain, and dimerization domain had little to no effect on the basal NF-κB promoter activity. Importantly, overexpression of DN-Mst1, which has been shown to inhibit endogenous Mst1 activity in various types of cells (15, 25), markedly suppressed TNF-α–induced NF-κB activation, as measured by both luciferase activity and EMSA (Figure 2, C and D). Mst1 is an upstream kinase of the JNK1/2 and p38 MAPK (27), which are involved in the activation of NF-κB. In human lung ECs, we found that Mst1 overexpression induced phosphorylation of JNK and p38 MAPK (Figure 2E), and pharmacological inhibition of JNK1/2 and p38 MAPK significantly attenuated Mst1-induced NF-κB activation in ECs (Figure 2F). Together, these results suggest that Mst1 induces NF-κB activation at least in part through targeting the JNK1/2 and p38 MAPK in lung ECs.

### Inhibition of Mst1 suppresses cytokine-induced lung EC activation.

**Figure 3:**
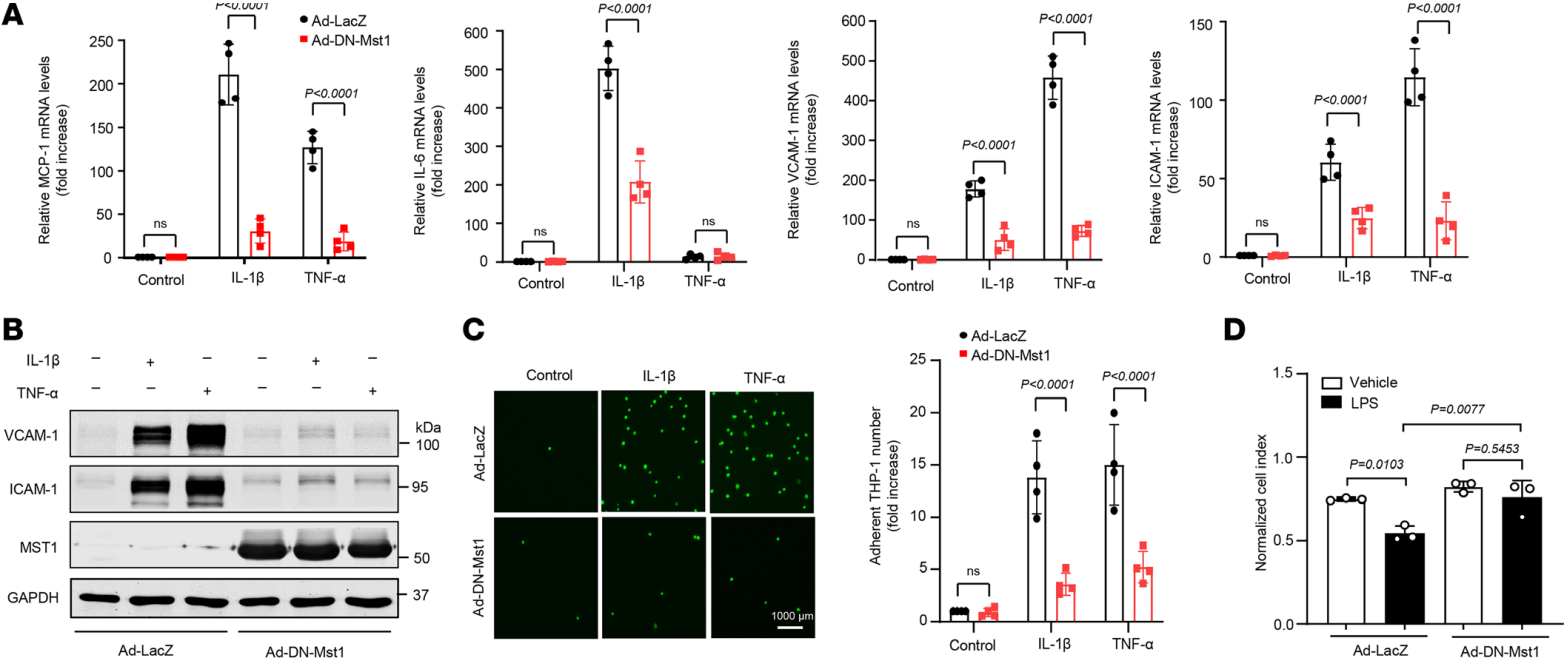
Overexpression of DN-Mst1 attenuates lung EC activation. Lung ECs were transduced with Ad-LacZ, or Ad-DN-Mst1 (MOI, 50). Forty-eight hours after adenovirus transduction, ECs were treated with or without 20 ng/mL TNF-α or 10 ng/mL IL-1β for 8 hours. The expression of MCP-1, IL-6, VCAM-1, and ICAM-1 was detected using qPCR (**A**) and by Western blot (**B**) using anti-VCAM-1 or anti-ICAM-1 antibodies. *n* = 4. (**C**) DN-Mst1 inhibits THP1 adherences to lung ECs. ECs were transduced with Ad-LacZ or Ad-Mst1 (MOI, 50). Forty-eight hours after adenovirus transduction, confluent lung ECs were then treated in the presence or absence of 20 ng/mL TNF-α or 10 ng/mL IL-1β for 8 hours and incubated with calcein-labeled THP1 cells for another 1 hour. Following washing, attached THP1 cells were visualized and counted with an inverted fluorescent microscopy. Scale bars: 1,000 μm. *n* = 4. (**D**) Lung ECs were seeded in the 16-well E-Plate at 3.5 × 10^4^ cells/well and incubated for 48 hours. Once the cells were confluent, the cells were then transduced with Ad-LacZ or Ad-DN-Mst1 at a MOI of 20. Twenty-four hours after virus transduction, the cells were starved with 1% FBS-ECM for 12 hours. Subsequently, the cells were treated with either vehicle (PBS) or LPS (10 μg/mL). Cell Index (CI) was then monitored at 8 hours. *n* = 3. Significance was determined by a 2-way ANOVA with Bonferroni’s post hoc test (**A**, **C** and **D**).

Activation of NF-κB is responsible for EC activation and the cytokine-induced expression of adhesion molecules, such as VCAM-1 and ICAM-1, which mediate the adhesion of monocytes to inflamed ECs (28). Thus, we examined the effects of Mst1 inhibition on the TNF-α– and IL-1β–induced expression of inflammatory molecules in human lung ECs. As shown in Figure 3A, adenovirus-mediated overexpression of DN-Mst1 substantially attenuated the TNF-α– and IL-1β–induced-expression of MCP-1, IL-6, ICAM-1, and VCAM-1, as determined by qPCR. Western blot demonstrated that both TNF-α– and IL-1β–induced-expression of ICAM-1 and VCAM-1 were also markedly suppressed in DN-Mst1–overexpressing ECs (Figure 3B). Monocyte adhesion to ECs is an important event in the initiation of vascular inflammation (28). Therefore, we examined the effect of overexpressing DN-Mst1 on THP-1 cell adhesion to the activated lung ECs. Stimulation of lung ECs with either TNF-α (20 ng/mL) or IL-1β (10 ng/mL) substantially increased THP-1 adhesion, which was markedly suppressed by approximately 85% in response to overexpressing DN-Mst1 (Figure 3C). Further, we found that overexpressing DN-Mst1 attenuated LPS-induced vascular permeability in human lung ECs (Figure 3D). Taken together, these results further suggest that Mst1 functions as a positive regulator of the cytokine-induced inflammatory responses in lung ECs.

### Mst1 deletion in ECs attenuates LPS-induced ALI in mice.

**Figure 4:**
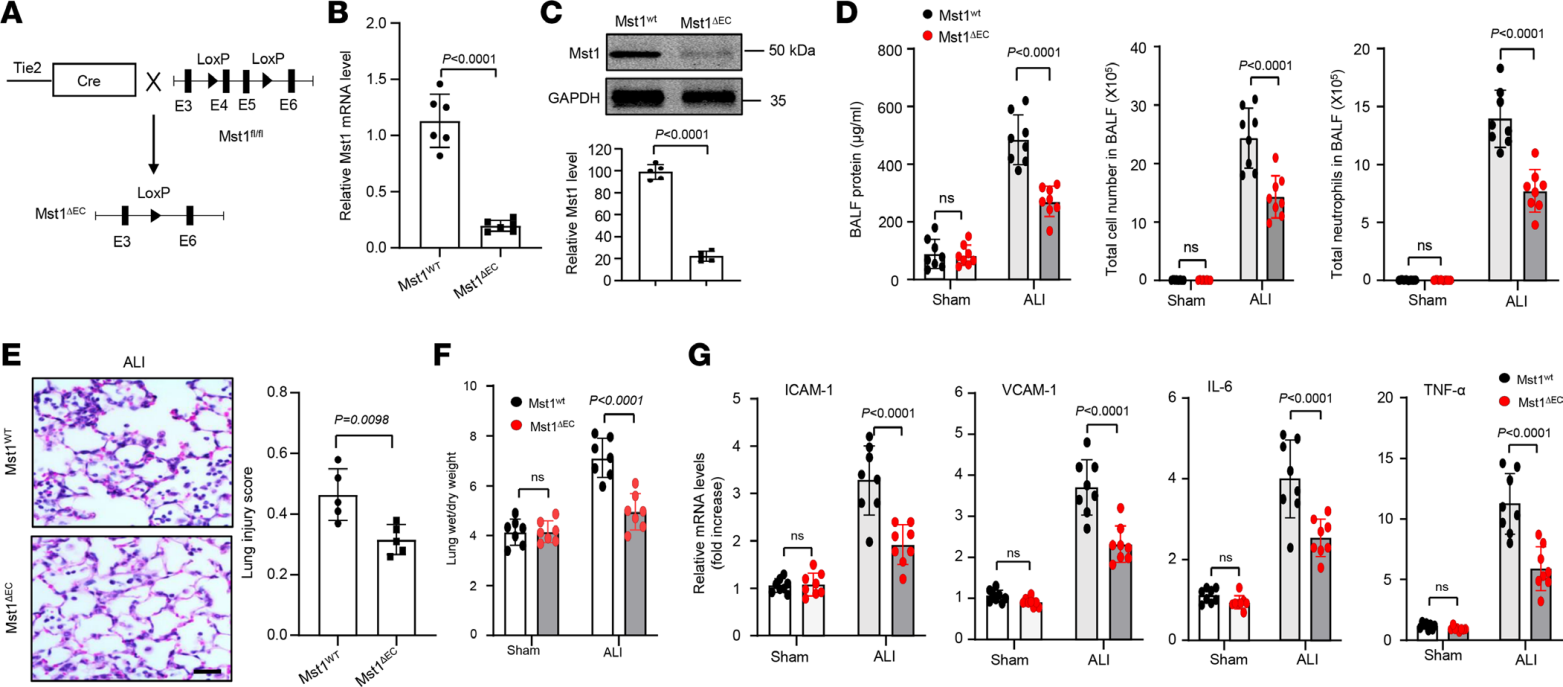
Endothelial deficiency of Mst1 attenuates LPS-induced lung injury in mice. (**A**) Strategy of generating Mst1 endothelial KO mice. (**B** and **C**) Expression of Mst1 in lung ECs isolated from Mst1^WT^ and Mst1^ΔEC^ mice was determined by qPCR and Western blot. *n* = 5–6. (**D**) Total cell and neutrophil counts and protein levels in bronchoalveolar lavage fluid (BALF) of Mst1^WT^ and Mst1^ΔEC^ mice after LPS instillation (*n* = 8). (**E**) Mst1^WT^ and Mst1^ΔEC^ mice were subjected to LPS instillation. Lung samples were harvested from mice at 24 hours after treatment. Representative H&E staining (*n* = 8 mice per group) of lung sections shows marked suppression of lung inflammatory injury in Mst1^ΔEC^ mice compared with Mst1^WT^ mice. Scale bars: 100 μm. *n* = 8. (**F** and **G**) Mst1^WT^ and Mst1^ΔEC^ mice were subjected to LPS instillation. Lung samples were harvested from mice at 24 hours after LPS treatment for measurement of lung wet/dry ratio and expression of cytokines and adhesion molecules were determined by qPCR. *n* = 8. Significance was determined by a Student’s 2-tailed *t* test (**B**, **C**, and **E**) and a 2-way ANOVA with Bonferroni’s post hoc test (**D**, **F**, and **G**).

To define the in vivo functional significance of Mst1 in EC activation, we generated endothelial Mst1 knockout mice (Mst1^fl/fl^/Cre^+/–^, Mst1^ΔEC^) by crossing Mst1^fl/fl^ mice (Mst1^wt^) with Tie2-Cre transgenic mice (Figure 4A). We observed Mst1^ΔEC^ mice for 1 year and did not appreciate any detectable phenotypic differences with Mst1^wt^mice. To further verify the KO efficacy of Mst1 in vasculature, we determined Mst1 expression by qPCR and Western blot in isolated lung ECs of Mst1^wt^ and Mst1^ΔEC^ mice. As shown in Figure 4, B and C, both Mst1 mRNA and protein levels in lung ECs were significantly decreased by nearly 80% in Mst1^ΔEC^ mice compared with Mst1^wt^ mice. To test the role of endothelial Mst1 in ALI responses, we instilled LPS in the airways of Mst1^wt^ and Mst1^ΔEC^. To quantify lung injury responses in these mice, we first measured the total number of inflammatory cells and total protein levels in the BALF. As shown in Figure 4D, endothelial-specific depletion of Mst1 markedly attenuated the total number of inflammatory cells and protein levels in the BALF after LPS exposure. Further, as shown in Figure 4, E–G, LPS-induced lung injury score, lung weight wet-to-dry ratio, and increased expressions of adhesion molecules and cytokines were substantially reduced in the lungs of Mst1^ΔEC^ compared with Mst1^wt^ mice. Together, these data suggest that endothelial Mst1 plays a pivotal role in LPS-induced ALI in mice.

### Pharmacological inhibition of Mts1 attenuates lung EC activation and ALI in mice.

**Figure 5:**
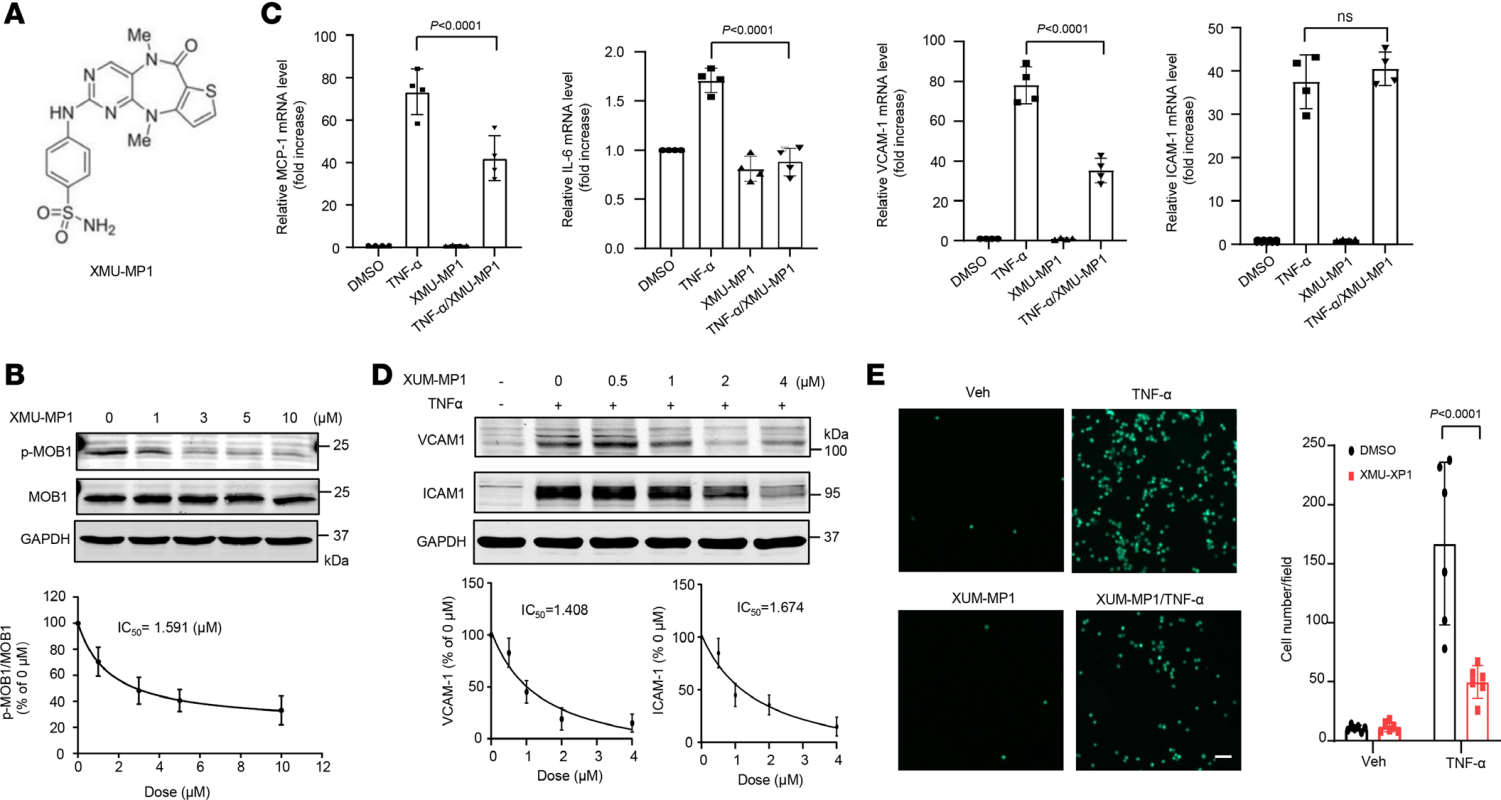
Pharmacological inhibition of Mst1 attenuates lung EC activation. (**A**) The structure of Mst1/2 inhibitor XMU-MP1. (**B**) Human lung ECs were treated with indicated concentrations of XMU-XP1 for 24 hours. Phosphorylation of MOB1 was determined by Western blot. (**C**) Lung ECs were pretreated with vehicle (DMSO) or 2 μmol/mL XMU-MP1 for 2 hours and then stimulated with TNF-α for 6 hours. The expression of MCP-1, IL-6, VCAM-1, and ICAM-1 was determined by qPCR. *n* = 4. Significance was determined by 2-way ANOVA with Bonferroni’s post hoc test. (**D**) Lung ECs were pretreated with vehicle (DMSO) or increasing concentration of XMU-MP1 for 2 hours and then stimulated with TNF-α for 12 hours. The expression of VCAM-1 and ICAM-1 was determined by Western blot. The expression of VCAM-1 and ICAM-1 was quantitated by densitometric analysis. IC_50_ was then calculated. *n* = 3. (**E**) Lung ECs were pretreated with vehicle (DMSO) or 2 μmol/mL XMU-MP1 for 2 hours and then treated in the presence or absence of 20 ng/mL TNF-α for 8 hours and incubated with calcein-labeled THP1 cells for another 1 hour. Following washing, attached THP1 cells were visualized and counted on an inverted fluorescent microscopy. Scale bars: 1,000 μm. *n* = 6. Significance was determined by a 2-way ANOVA with Bonferroni’s post hoc test (**C** and **E**).

To further explore the therapeutic capacity of targeting Mst1 for the treatment of ALI, we tested whether pharmacological inhibition of Mst1 could attenuate ALI in mice. Since specific inhibitors of Mst1 are not available, we examined the effects of XMU-MP1 (Figure 5A), which has been shown to selectively block both Mst1 and Mst2 (29), on endothelial activation. We first examined the inhibitory effects of XMU-MP1 on Mst1 in ECs, as determined by measuring phosphorylation of its substrate MOB1 (Mps One Binder homolog A) by Western blot. As shown in Figure 5B, XMU-MP1 dose-dependently inhibited phosphorylation of MOB1 in human lung ECs with IC_50_ of 1.591 μmol/L. We then determined the effects of XMU-MP1 on the expression of cytokines and adhesion molecules in ECs. To this end, we pretreated human lung ECs with 2 μmol/L XMU-MP1 for 2 hours before the stimulation of ECs with TNF-α for 12 hours. We then determined the expression of cytokines and adhesion molecules by qPCR. As shown in Figure 5C, TNF-α–induced increased expression of MCP-1, IL-6, and VCAM-1 was significantly inhibited by XMU-MP1, whereas ICAM-1 mRNA levels were not similarly affected. However, XMU-MP1 significantly inhibited TNF-α–stimulated protein expression of VCAM-1 and ICAM-1, with IC_50_ of 1.408 μmol/L and 1.674 μmol/L respectively, and this associated with marked suppression of monocyte adhesion to activated ECs (Figure 5, D and E).

**Figure 6:**
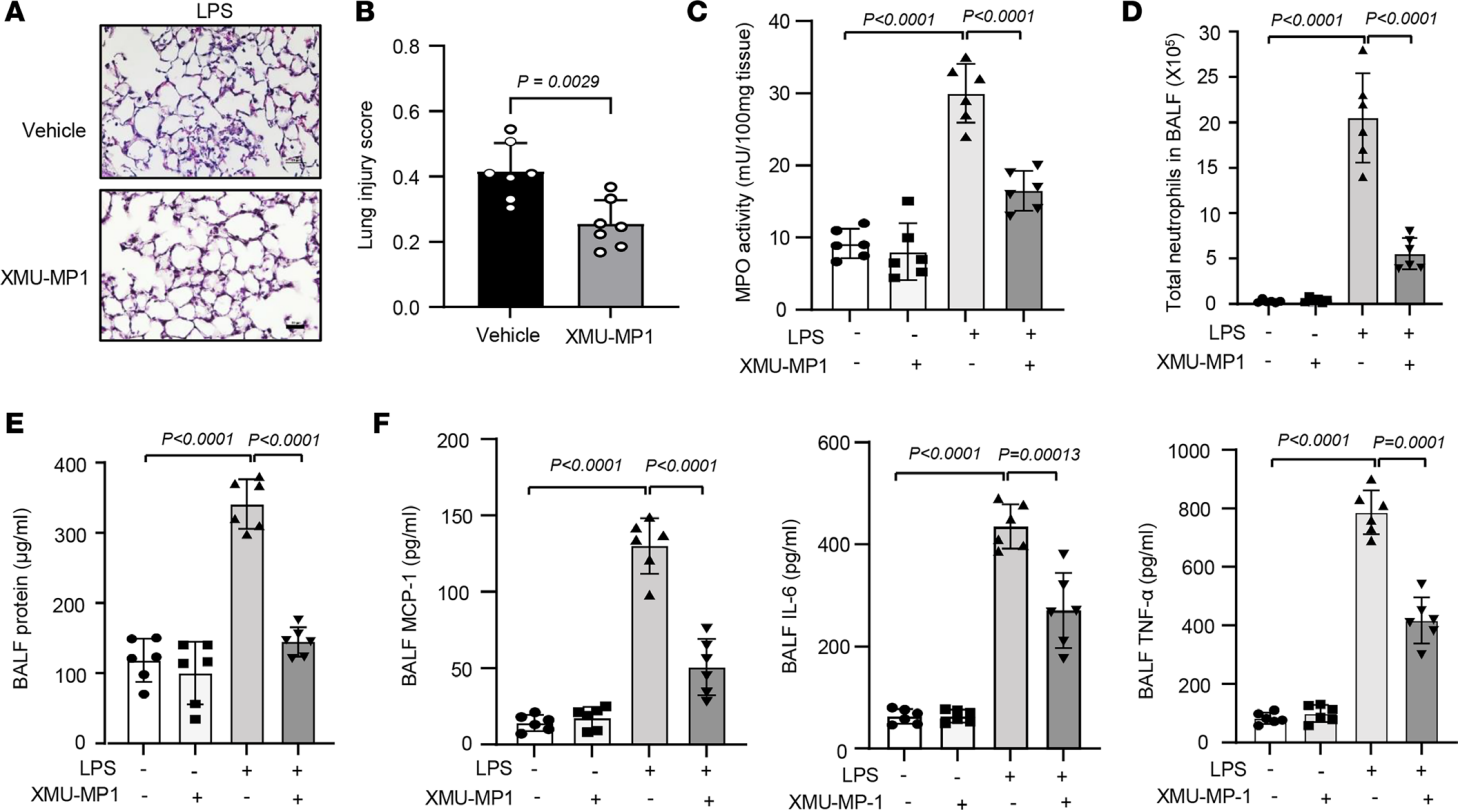
Pharmacological pretreatment to inhibit Mst1 attenuates LPS-induced ALI in mice. WT C57BL6J mice were pretreated with either vehicle or XMU-MP1 (4 mg/kg, IP) for 2 hours and then subjected to LPS instillation. 24 hours after LPS instillation, lung tissues were collected for H&E staining (**A**) and lung injury score analysis (**B**). The MPO activity (**C**), neutrophils (**D**), and the total protein (**E**), and the levels of MCP-1, IL-6, and TNF-α (**F**) in bronchoalveolar lavage fluid (BALF) at baseline and after LPS stimulation were determined. Significance was determined by a Student’s 2-tailed *t* test (**B**) and a 2-way ANOVA with Bonferroni’s post hoc test (**C**–**F**).

**Figure 7:**
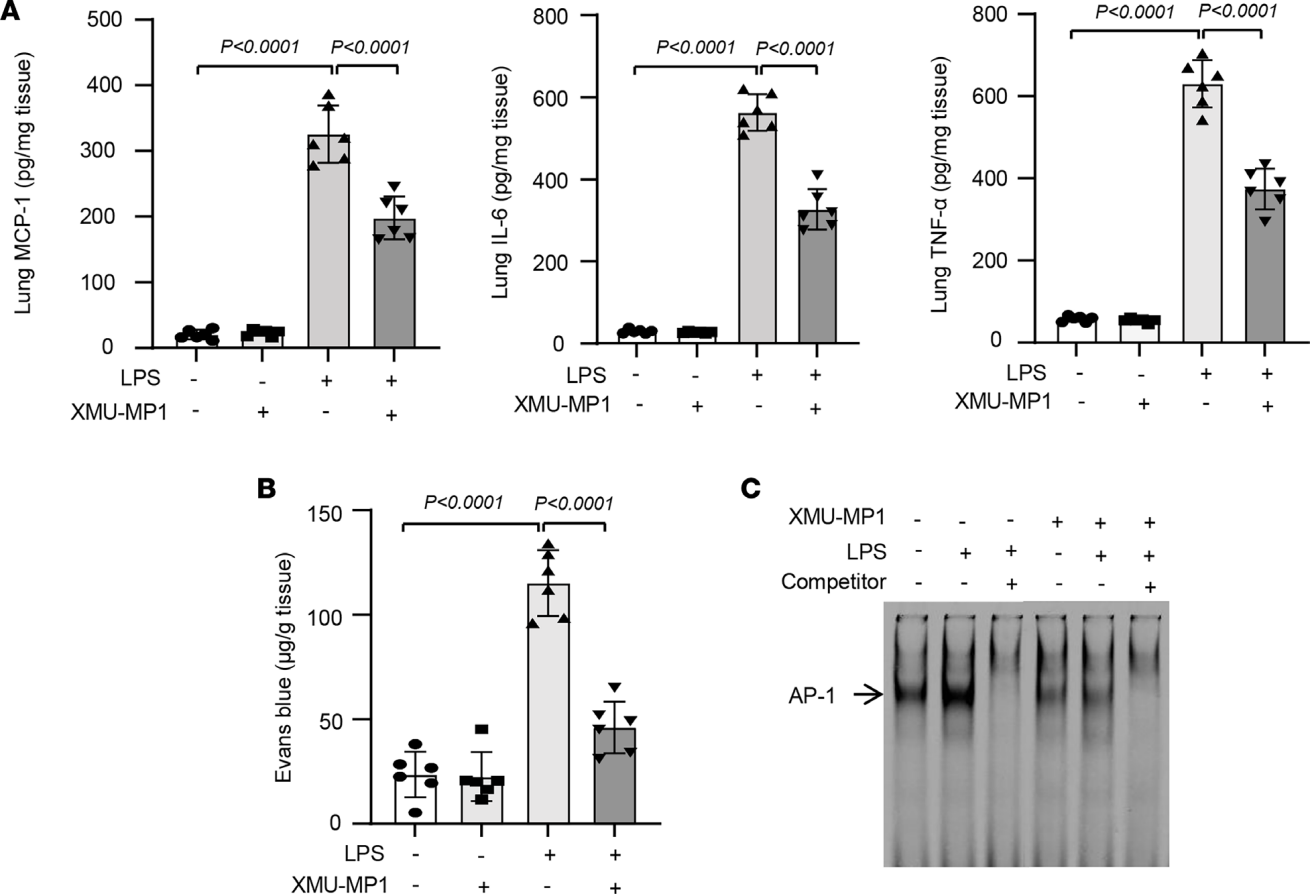
Pharmacological pretreatment to inhibit Mst1 attenuates LPS-induced lung inflammation in mice. (**A**) WT C57BL6J mice were pretreated with either vehicle or XMU-MP1 (4 mg/kg, IP) for 2 hours and then subjected to LPS instillation. Twenty-four hours after LPS instillation, lung tissues were collected and the levels of MCP-1, IL-6, and TNF-α in lung samples were determined by ELISA. (**B**) Evans blue dye extravasation in lung of vehicle and XMU-MP-1 treated mice were determined. *n* = 6/group. (**C**) Twenty-four hours after LPS instillation, nuclear fractions from mouse lung tissues were isolated to determine the AP-1 activation by EMSA. 50-fold excess of the unlabeled oligonucleotide as a competitor was included to show the binding specificity. Significance was determined by a 2-way ANOVA with Bonferroni’s post hoc test (**A** and **B**).

**Figure 8:**
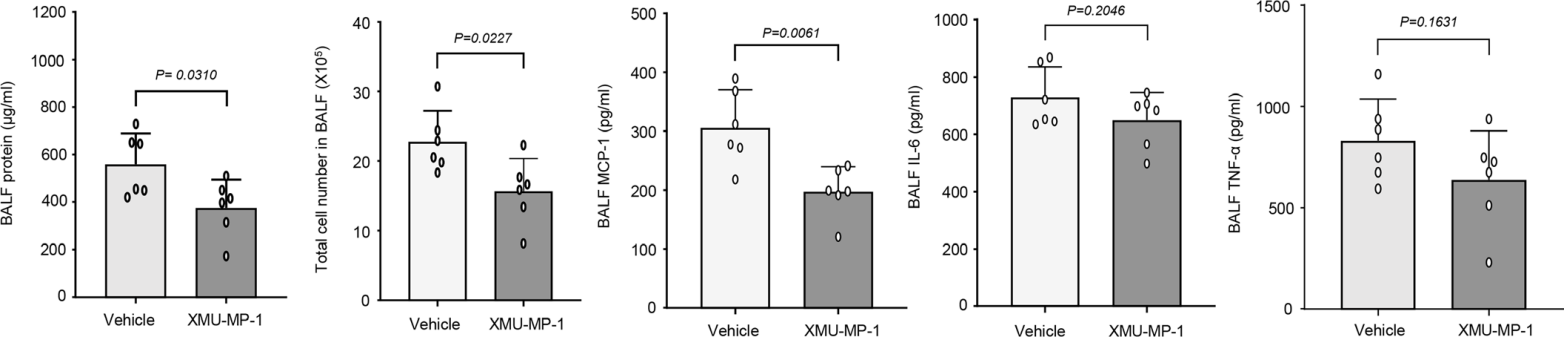
Therapeutic effect of Mst1 inhibitor on ALI in mice. C57BL/6J mice (8–12 weeks) were subjected to LPS instillation (100 μg/mouse). XMU-MP-1 (i.p. 10 mg/kg) was given to the mice at 0.5 hour and 12 hours after LPS instillation. 24 hours after LPS instillation, the levels of total protein, inflammatory cells, MCP-1, and IL-6 in BALF were determined. *n* = 6. Significance was determined by a Student’s 2-tailed *t* test.

To define the in vivo therapeutic effects of XMU-MP1, we again employed a LPS-induced murine ALI model (30). In this regard, mice were pretreated with XMU-MP1 (4 mg/kg, IP) for 2 hours before LPS instillation. Twenty-four hours after LPS administration, BALF and lung tissues were collected to evaluate the therapeutic effects of XMU-MP1 on ALI. As shown in Figure 6, A–D, LPS-induced lung injury and neutrophil infiltration were inhibited by XMU-MP1 treatment. Treatment of LPS increased BALF neutrophil and protein levels in vehicle-treated mice, which were markedly inhibited in XMU-MP1 treated mice (Figure 6, C–E). Likewise, XMU-MP1 treatment substantially attenuated LPS-induced expression of chemokines and cytokines in BALF, as determined by ELISA (Figure 6F). Further, LPS-induced expression of MCP-1, IL-6, and TNF-α was inhibited by XMU-MP1 treatment (Figure 7A). Pulmonary vascular leakage, as determined by Evans blue dye extravasation, was similarly decreased in XMU-MP1–treated mice (Figure 7B). Mechanistically, we found that XMU-MP1 inhibited the LPS-induced activation of activator protein-1 (AP-1) as determined by EMSA at 2 hours after LPS instillation (Figure 7C). Further, our pilot studies showed that administration of XMU-MP1 after LPS instillation significantly decreased total protein levels, inflammatory cells, and MCP1 levels in BALF, while the levels of IL-6 and TNF-α only trended toward reduced levels (Figure 8). Future studies will be focused on optimizing treatment schedules and testing therapeutic efficacy in other lung injury models, such as bacterial pneumonia models. Taken together, these data suggest that pharmacological inhibition of Mst1/2 can effectively prevent EC activation and LPS-induced ALI in mice and points to a role for Mst1 inhibitor therapies in the management of inflammatory vascular diseases of the lung.

## Discussion

Mst1/2 are core members of the Hippo pathway (31) and have been implicated in the pathobiology of various diseases (32). For instance, recent studies have demonstrated that Mst kinase is critically involved in the development of several lung diseases, such as pulmonary artery hypertension (PAH) (33), lung fibrosis (34, 35), pneumonia (36), and lung regeneration (20). However, the importance of Mst kinase in controlling ALI has not been investigated. In the present study, we show that Mst1 is activated in lung ECs in response to TNF-α stimulation and that activation contributes to heightened endothelial responses by increasing NF-κB signaling. Moreover, we demonstrate that genetic and pharmacological approaches to inhibiting Mst1 activity render mice more resistant to LPS-induced lung injury, supporting the notion of Mst1 directed therapies for the treatment of human disease.

The lung endothelium serves as a formidable barrier against the influx of cells and the leakage of proteins and other circulating materials in the lung (2). It has been increasingly recognized that the pulmonary endothelium plays a pivotal role in orchestrating inflammatory responses to pulmonary insults (2). In response to a range of pathological insults, such as hypoxia, cytokines, chemokines, and bacterial endotoxins, the lung endothelium produces a variety of cytokines and leucocyte adhesion molecules including intracellular adhesion molecule-1 (ICAM-1), vascular cell adhesion molecule-1 (VCAM-1) and E-selectin, which helps to recruit inflammatory cells from the circulation and support their transendothelial migration into the lung parenchyma (7). Thus, therapeutic strategies to suppress endothelial inflammation are anticipated to ameliorate ALI and other inflammatory vascular conditions of the lung. At the molecular level, activation of NF-κB is fundamentally important in driving endothelial inflammation and the upregulation in expression of EC adhesion molecules. Upstream regulators of NF-κB in ECs have been poorly identified and require further investigation. Mst1 is an important component of the hippo/yap pathway that has been implicated in organ size control and tissue regeneration and self renewal (31). Mst1 is proteolytically cleaved by caspase to a 34–36-kDa N-terminal catalytic fragment, which increases Mst1 kinase activity and nuclear translocation (37, 38). In addition to caspase cleavage, Mst1 phosphorylation at Thr183 has been proposed to contribute to kinase activation (23). In accordance with these findings, we found that Mst1 phosphorylation at Thr183 was significantly increased in both inflamed lung ECs and mouse lung tissues after LPS exposure, further highlighting the critical role of Mst1 activation in the development of inflammatory lung disease.

Recently, several studies have implicated the regulatory roles of Mst1 in NF-κB activation (16, 39–42). Depending on the cell type, Mst1 has been shown to promote or inhibit NF-κB activation. For instance, Mst1 has been shown to promote NF-κB activation in brain tissues, cardiomyocytes, and skin lesions of psoriasis (16, 41, 43). On the other hand, Mst1 activation inhibits NF-κB activation in fibroblasts and cancer cells (40, 44). The underlying reasons for these opposing effects have not been explained. In this study, we found that Mst1 activation promotes NF-κB activation in lung ECs and that overexpression of DN-Mst1 suppressed NF-κB activity, supporting the notion that endogenous Mst1 regulates the transcriptional activity of NF-κB in lung ECs. We and others have shown that Mst1 is an upstream kinase of the JNK and p38 MAPK pathways (45, 46), which have been shown to induce NF-κB activation in a wide range of cells (47). Consistent with these observations, we found that Mst1 induces the activation of the JNK and p38 MAPK pathways in lung ECs and that pharmacological inhibition of JNK and p38 MAPK markedly attenuated Mst1-induced NF-κB activation in lung ECs as well as LPS-induced AP-1 activation in mouse lung tissues, further suggesting the role of the JNK and p38 MAPK in Mst1-induced NF-κB activation and inflammation in lung ECs. Further, Mst1/2 has been shown to be an important upstream kinase implicated in the negative regulation of YAP activation (20), and activation of YAP in lung epithelial cells has recently been shown to inhibit endotoxemic ALI in mice (48). Whether Mst1 regulates NF-κB activation through the YAP pathway in lung ECs warrants further investigation. Another limitation of the present study is that Tie2-Cre may cause Mst1 deletion not only in ECs but also in hematopoietic cells. The potential role of Mst1 from hematopoietic cells in LPS-induced ALI will be addressed in our future studies.

XMU-MP-1 has been identified as a potent and specific inhibitor of Mst1/2 kinases and it has been shown to inhibit Mst1/2 activities in various in vitro and in vivo studies (29, 49, 50). In this study, we demonstrated that pharmacological inhibition of Mst1 by XMU-MP-1 dramatically suppressed lung inflammation and vascular leakage to LPS in mice. Further, our in vitro studies showed that XMU-MP-1 dramatically attenuated TNF-α–induced expression of cytokines, chemokines, and VCAM-1. Interestingly, this treatment barely affected ICAM-1 mRNA levels, but significantly inhibited ICAM-1 protein expression in lung ECs, suggesting that XMU-MP-1 may also regulate ICAM-1 expression at posttranslational levels, which needs further investigation. Consistent with our results, XMU-MP-1 has been recently shown to inhibit endotoxemic ALI in mice through activating YAP in lung epithelial cells (48). Moreover, XMU-MP-1 has been shown to inhibit both Mst1 and Mst2 activation. Although our Mst1 EC-specific KO mice exhibit a markedly decreased susceptibility to LPS-induced EC activation and lung injury, we cannot completely rule out the potential involvement of Mst2 in this process. This limitation needs to be addressed in our future studies. In this study, we found that transduction of Ad-DN-Mst1 or inhibition with XMU-MP1 has no effect on the morphology of the lung ECs, suggesting that Mst1-mediated EC death may not contribute to LPS-induced lung injury. The decision to administer XMU-MP-1 prior to the induction of ALI points to a role for Mst1 inhibitor treatments in the prevention of ALI. We speculate that this could be important for patients at high risk for ALI and ARDS. Future studies will be needed to optimize the therapeutic regimen and test the efficacy of Mst1 inhibitors in other lung injury models such as a bacterial pneumonia model.

In summary, our results provide both in vitro and in vivo evidence to demonstrate that Mst1 is a critical regulator of lung endothelial activation and inflammation through activation of the NF-κB pathway. Targeted inhibition of Mst1 may represent a novel therapeutic strategy for the prevention and treatment of inflammatory lung diseases such as ALI and ARDS.

## Methods

### Sex as a biological variable.

Both male and female mice were used to account for sex as a biological variable.

### Cell culture.

Human lung microvascular ECs were purchased from ATCC and cultured in EBM-2 medium supplemented with EGM-2 BulletKit (Lonza). THP-1 and U937 cells were purchased from ATCC and cultured in RPMI medium 1640 (Corning, 10-040-CM). EA.hy926 cells were purchased from ATCC and cultured in Dulbecco Modified Eagle Medium (DMEM, Corning, 10013CV DMEM). All medium was supplemented with FBS (Gibco, 1082147) and penicillin/streptomycin (Corning, 30-002-CI).

### Antibodies and reagents.

Anti-Mst1 (#14946, 1:500 dilution), anti-p65 (#8242, 1:1,000 dilution), anti-p38 (#9212, 1:1,000 dilution), anti-phospho-p38 (#9211, 1:1,000 dilution), anti-Erk1/2 (#4695, 1:1,000 dilution), anti-phospho-Erk1/2 (#4370, 1:1,000 dilution), anti-JNK1/2 (#9252, 1:1,000 dilution), anti-phospho-JNK1/2 (#9251, 1:1,000 dilution), anti-p-MOB1 (#8699, 1:1,000 dilution), and anti-MOB1 (#13730, 1:1,000 dilution) antibodies were purchased from Cell Signaling Technology. Anti-phospho-Mst1 (AP0906, 1:500 dilution) antibody was acquired from ABclonal Technology. Anti-ICAM1 (sc-7891, 1:800 dilution), anti-VCAM1 (sc-13160, 1:300 dilution), and anti-GAPDH (sc-32233, 1:1,000 dilution) antibodies were from Santa Cruz Biotechnology. IRDye 680RD donkey anti-rabbit (926-68073, 1:10,000 dilution) and 800CW goat anti-mouse (926-32210, 1:10,000 dilution) antibodies were obtained from LI-COR Bioscience. LPS (sc-3535) was acquired from Santa Cruz Biotechnology. Recombinant Human TNF-α (300-01A) and IL-1β (200-01B) were acquired from PeproTech. DAPI (4′,6-diamidino-2-phenylindole) (D1306) was purchased from Invitrogen. XMU-MP1 (#22083, Cayman) was dissolved in 2% DMSO + 30% PEG 300 + 2% Tween 80 + ddH2O at 5 mg/mL for in vivo studies. JNK inhibitor (SP600125, #HY-12041) and p38 MAPK inhibitor (SB203580, #HY-10256) were purchased from MedChemExpress.

### Mice.

Mst1/2 floxed mice (STK3^fl/fl^/STK4^fl/fl^ mice were purchased from The Jackson Laboratory (Stock # 017635, JAX) and crossed with C57BL/6J to generate Mst1 floxed mice (Mst1^fl/fl^) with exons 4 and 5 of Mst1 flanked by loxP sites (51). Mst1^fl/fl^ mice were then interbred with Tie2-Cre mice (Stock number: 008863, the Jackson Laboratories) to generate endothelial Mst1-KO mice (Mst1^ΔEC^). All mice were genotyped by PCR. The genomic DNA isolated from mice tail biopsies was analyzed by PCR to detect Mst1 WT allele (product size 303 bp), loxP-flanked allele (450 bp), and Tie2-Cre allele (100 bp). Genotyping primers for Mst1 allele were 5′-AGTGTTGGCTCTTGATTTTCCT-3′ (forward) and 5′-CAGGGCTAGAGTGAAACCTTG-3′ (reverse), and genotyping primers for Cre allele were 5′-GCGGTCTGGCAGTAAAAACTATC -3′ (forward) and 5′-GTGAAACAGCATTGCTGTCACTT -3′ (reverse). All mice were on the C57BL/6J background and maintained under specific pathogen–free conditions at 22°C with a 12-hour light/ 12-hour dark cycle. Age-matched littermates (8–12 weeks old) were used for the experiments. All animal protocols were approved by the Institutional Animal Care and Use Committee at Thomas Jefferson University before performing any studies.

### Murine ALI model.

ALI was induced as described previously (30). In brief, anesthetized mice (8–12 wk, 20–25 g) were suspended from a sloped board, and a 1-time dose of LPS (100 μg per mouse) was instilled in the posterior oropharyngeal space. For preventive studies, XMU-MP1 (4 mg/kg, IP) was administrated 2 hours before LPS instillation. For treatment studies, XMU-MP1 (10 mg/kg, IP) was administrated at 0.5 hours and 12 hours after LPS instillation. Twenty-four hours after LPS administration, lung tissues were harvested for further analysis. To avoid experimental bias, genotypes and experimental grouping were blinded to the observers.

### Bronchoalveolar lavage.

Bronchoalveolar lavage (BAL) was performed by cannulating the trachea with a blunt 22-gauge needle and instilling the same 1 mL of sterile PBS back and forth in the airways 3 times as described previously (30). Total cell counts were performed using a TC20 automated cell counter (Bio-Rad Laboratories), and differential cell counts were performed after cytocentrifuging cells on glass slides and staining with HEMA 3 (Thermo Fisher Scientific). Total protein levels were determined as previously described (30). The activity of the enzyme myeloperoxidase (MPO), a marker of polymorphonuclear neutrophil primary granules, was determined in a lung fragment (upper right lobe) after pulmonary lavage, as described previously (52).

### ELISA.

MCP-1, TNF-α, and IL-6 levels were quantified in BALF and lung tissues using commercially available DuoSet ELISA kits (R&D Systems) according to manufacturer’s instructions and published protocols (30). Briefly, Nalgene Nunc Maxisorp plates were coated overnight with antibodies to MCP-1 (4 μg/mL), TNF-α (4 μg/mL), and IL-6 (4 μg/mL) and the next morning plates were washed and blocked for 2 hours. Samples were added to the wells at various dilutions, followed by incubation with detection antibody for 2 hours. Plates were subsequently washed, and streptavidin-horseradish peroxidase conjugate antibody was added to each well for 20 minutes. This was followed by an additional wash step, before plates were incubated with 3,3′,5,5′-tetramethylbenzidine (TMB) substrate solution (R&D Systems). Enzymatic reactions were quantified by measuring absorbance at 450 nm using a standard plate reader (Biotek Instrument).

### Lung histology.

Lung histology was performed on paraformaldehyde-fixed tissues embedded in paraffin wax. Sections (5 μm) were placed on positively charged glass slides, and tissues were deparaffinized before undergoing H&E staining. Tissues were visualized using standard light microscopy (24).

### Pulmonary microvascular permeability.

Lung permeability was assessed by quantifying the extravasation of Evans blue dye (E2129, Sigma-Aldrich). Evans blue (20 mg/kg; Sigma-Aldrich) was injected intravenously 2 hours before euthanasia. Before lungs were extracted from the thorax, the pulmonary vasculature was gently perfused by slowly injecting saline in the spontaneously beating right ventricle. Extraction of Evans blue dye was performed by incubating tissues at 65°C with formamide (2 mL/g tissue) overnight. Lung tissues were then centrifuged (12,000*g* for 30 minutes), and Evans blue dye concentration in supernatant was determined spectrophotometrically by measuring absorption at 620 nm and correcting for the presence of heme pigments: A620 (corrected) = A620 − (1.426 × A740 − 0.030) where A620 is absorbance at 620 nm and A740 is absorbance at 740 nm. The lung wet-to-dry (W/D) weight ratio was used as an index of lung water accumulation after the instillation of LPS. To measure the total amount of lung water, the animals were dissected under deep sevoflurane anesthesia, and the lung weight was measured immediately after its excision (wet weight). The lung tissue was then dried in an oven at 60°C for 5 days and reweighed as dry weight. The W/D weight ratio was calculated by dividing the wet by the dry weight.

### Murine lung EC Isolation.

The lungs from WT and Mst1-KO mice (at 7–9 days of age, 3 per group) were dissected into single lobes and incubated in DMEM containing collagenase solution. The cell suspension was purified with anti-CD31–coated magnetic beads (Invitrogen) and cultured in extracellular matrix medium (ScienCell) supplemented with 1% penicillin–streptomycin solution, 1% EC growth stimulant, and a 20% FBS BulletKit (Lonza) as described previously (24).

### Monocyte adhesion assay.

Confluent lung ECs transduced with Ad-LacZ or Ad-DN-Mst1 were treated with 10 ng/mL IL-1β or 20 ng/mL TNF-α for 8 hours to induce the expression of adhesion molecules before adhesion assay. THP-1 cells were labeled with calcein-AM (Invitrogen) according to the manufacturer’s instruction. After lung ECs were stimulated and washed, 2.5 × 10^5^ calcein-labeled THP-1 cells were added to each well and allowed to interact for 60 minutes at 37°C as we described previously (28). Unbound cells were removed by gently washing with complete medium, and the number of attached THP-1 cells was counted on an inverted fluorescent microscope.

### EC permeability assay.

Endothelial permeability was determined by cellular impedance using xCELLigence Real-Time Cell Analysis (RTCA). Briefly, human pulmonary artery ECs (HPAECs) were seeded in the 16-well E-Plate at 3.5 × 10^4^ cells/ well and incubated for 48 hours. Once the cells were confluent (Cell Index reaches plateau phase), the cells were transduced with Ad-LacZ or Ad-DN-Mst1 at a MOI of 20. On the next day, the cells were starved with 1% FBS-ECM for 12 hours. Subsequently, the cells were treated with either vehicle or LPS at 10 μg/mL. Cell Index (CI) was monitored at 8 hours after addition of LPS.

### Immunofluorescence staining.

Lung ECs were fixed and sequentially incubated with primary antibodies and appropriate fluorescent-labeled secondary antibodies. Images were visualized using an Olympus IX70 epifluorescence microscope as previously described (53).

### Construction of adenoviruses.

Adenoviruses harboring WT Mst1 (Ad-Mst1) and DN Mst1 (Ad-DN-Mst1) were made using AdMax (Microbix), as previously described (54). The viruses were propagated in AD-293 cells and purified using CsCl2 banding, followed by dialysis against 20 mmol/L Tris-buffered saline with 10% glycerol. Titering was performed on AD-293 cells using Adeno-X Rapid Titer kit (Clontech) according to the instructions of the manufacturer (25).

### Quantitative real-time PCR.

Total RNA was extracted from human lung ECs and mouse lung tissues using TRIZOL reagent kit (Invitrogen). The cDNA was generated using iScript cDNA synthesis kit (170-8891; Bio-Rad). Real-time PCR analysis was performed with Power SYBR Green PCR Master Mix (4367659; Life Technologies) by a StepOne Plus system (Applied Biosystems). GAPDH RNA was used as an internal control. The relative difference was expressed as the fold matched control values calculated with the efficiency-corrected 2^–ΔΔCt^ method. The primer list for quantitative RT-PCR is provided in the Supplemental Table 1.

### Western blot analysis.

Western blot analysis was performed as we described previously (53). In brief, cell or tissue lysates were resolved by SDS-PAGE and transferred to nitrocellulose membrane (Bio-Rad Laboratories, USA). Blots were blocked with 5% nonfat milk in PBS, then developed with 5% PBST (PBS with 0.1% Tween20) and diluted primary antibodies followed by incubating with either IRDye 700 or 800–labeled secondary antibodies and then visualized using Odyssey Infrared Imaging System (Li-Cor, Lincoln, NE). The intensity of the bands was quantified by using the Odyssey software.

### Luciferase reporter assay.

EC line EA.hy926 cells were seeded in 12-well plates and incubated overnight. The cells were transfected with 100 ng of NF-κB firefly luciferase reporter plasmid p(NF-κB)_3_-Luc, which contains three copies of the NF-κB consensus driving expression of the firefly luciferase reporter gene (Stratagene) and 10 ng of Renilla luciferase reporter plasmid pRL-RSV (Promega), in the presence or absence of indicated expression vectors using FuGene 6 transfection reagent (Promega). Thirty-six hours after transfection, cells were treated with or without indicated stimuli for 8 hours, then directly lysed in the lysis buffer (Promega). Cell lysates (20 μL) were assayed for luciferase activity using the dual Luciferase Assay System (Promega, USA), according to manufacturer instructions. We used Renilla luciferase as a control for transfection efficiency. Firefly luciferase activity was normalized for transfection efficiency by corresponding Renilla luciferase activity (28).

### Electrophoretic mobility shift assay.

Electrophoretic mobility shift assay (EMSA) was performed as previously described (55). Briefly, the oligonucleotides corresponding to the consensus sequence of NF-κB (5′- AGTTGAGGGGACTTTCCCAGGC-3′) and AP1 (5′-CGCTTGATGACTCAGCCGGAA-3′) were synthesized and labeled with IRDye 700 (IDT). EMSA was performed with Odyssey IRDye 700 infrared dye–labeled double-stranded oligonucleotides coupled with the EMSA buffer kit (Thermo Fisher Scientific) as we described previously (53, 55). Briefly, 5 μg of nuclear extract was incubated with 1 μl of IRDye 700 infrared dye labeled double-stranded oligonucleotides, 2 μL of 10 × binding buffer, 2.5 mM DTT, 0.25% Tween-20 and 1 μg of poly (dI-dC) in a total volume of 20 μL for 20 minutes at room temperature in the dark. The specificity of the binding was examined using competition experiments, where 50-fold excess of the unlabeled oligonucleotides were added to the reaction mixture for 30 minutes. The gel supershift assay was performed by adding p-65 antibody (Cell Signaling) prior to the addition of the fluorescently labeled probe. Sample proteins were separated on a 4% polyacrylamide gel in 0.5 × Tris-borate-EDTA running buffer for 60 minutes at 70 V. The gel was scanned by direct infrared fluorescence detection on the Odyssey imaging system (LI-COR Bioscience).

### Statistics.

Data were presented as the mean ± SD. Normality of distribution was assessed by using Shapiro-Wilk test. Differences between groups with normally distributed data were analyzed using a 2-sided unpaired *t* test (for 2 groups of data), 1-way ANOVA followed by Tukey’s post hoc test (for 3 or more groups of data). 2-way ANOVA coupled with Tukey’s post hoc test was applied for 2 independent variables. For nonnormal distribution or sample number less than 6, 2-tailed Mann-Whitney test (2 groups) or Kruskal-Wallis test (multiple groups) was performed, followed by Dunn’s post hoc analysis. Values of *P* < 0.05 were considered statistically significant.

### Data availability.

Values for all data points in graphs are reported in the Supporting Data Values file.

## Author contributions

ZFG, NT, CL, CZ, LH, DC, and MXZ performed experiments. ZFG, NT, CL, CZ, RS, and JS designed experiments and analyzed and interpreted data. ZFG, RS, and JS wrote the paper. All authors edited the paper.

## Supplementary Material

Supplemental data

Unedited blot and gel images

Supporting data values

## Version 1 09/10/2024

Electronic publication
