# Supplementary material for: Inhibiting endothelial cell Mst1 attenuates acute lung injury in mice
Source: JCI Insight. 2024 Sep 10;9(17):e178208. doi: 10.1172/jci.insight.178208 (PMC11385092; doi:10.1172/jci.insight.178208)
Supplement: Supplemental data [file jciinsight-9-178208-s174.pdf]

Supplemental data

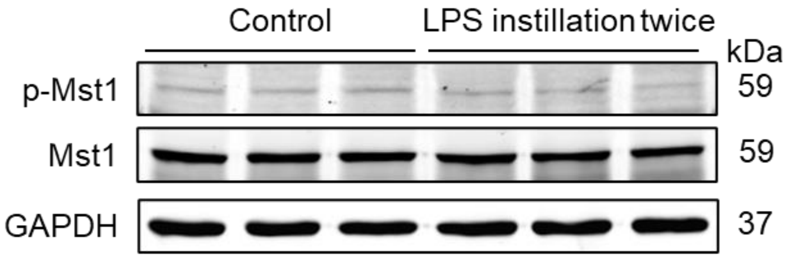

Fig. S1. Effect of repeated LPS instillation on Mst1 activation. Wildtype mice were anesthetized and instilled with 100 μg of LPS in posterior oropharyngeal space. 6 hrs after the first LPS administration, the mice were instilled with the second dose of LPS. 2 hrs after the second LPS administration, the whole lung tissues were collected for the determination of p-Mst1, Mst1, and GAPDH by western blot.

Supplemental Table I: Summary of the real-time PCR Primer sets used

**Primer**	**Sequence (5'-3')**
Human MCP-1	S: TCATAGCAGCCACCTTCATTC<br>AS:CTCTGCACTGAGATCTTCCTATTG
Human IL-6	S: GTACATCCTCGACGGCATCTC<br>AS:GGTTCAGGTTGTTTTCTGCCA
Human VCAM-1	S: GGCAGAGTACGCAAACACTT<br>AS: GGCTGTAGCTCCCCGTTAG
Human ICAM-1	S: CCGGAAGGTGTATGAACTGA<br>AS: GGCAGCGTAGGGTAAGGTT
Mouse IL-6	S: TAGTCCTTCCTACCCCAATTTCC<br>AS: TTGGTCCTTAGCCACTCCTTC
Mouse Mst1	S: CCGAGATATCAAGGCGGGAA<br>AS: GTTGACCTGCGACTCCAAAG
Mouse TNF-α	S: CCCTCACACTCAGATCATCTTCT<br>AS: GCTACGACGTGGGCTACAG
Mouse VCAM-1	S: AGTTGGGGATTCGGTTGTTCT<br>AS: CCCCTCATTCCTTACCACCC-3'
Mouse ICAM-1	S: GTGATGCTCAGGTATCCATCCA<br>AS: CACAGTTCTCAAAGCACAGCG-3
Human GAPDH	S: ACAACTTTGGTATCGTGGAAGG<br>AS: GCCATCACGCCACAGTTTC
Mouse GAPDH	S: AGGTCGGTGTGAACGGATTTG<br>AS: TGTAGACCATGTAGTTGAGGTCA
