# Supplementary figures and images for: Inhibiting endothelial cell Mst1 attenuates acute lung injury in mice
Source: JCI Insight. 2024 Sep 10;9(17):e178208. doi: 10.1172/jci.insight.178208 (PMC11385092; doi:10.1172/jci.insight.178208)

Fig.1A

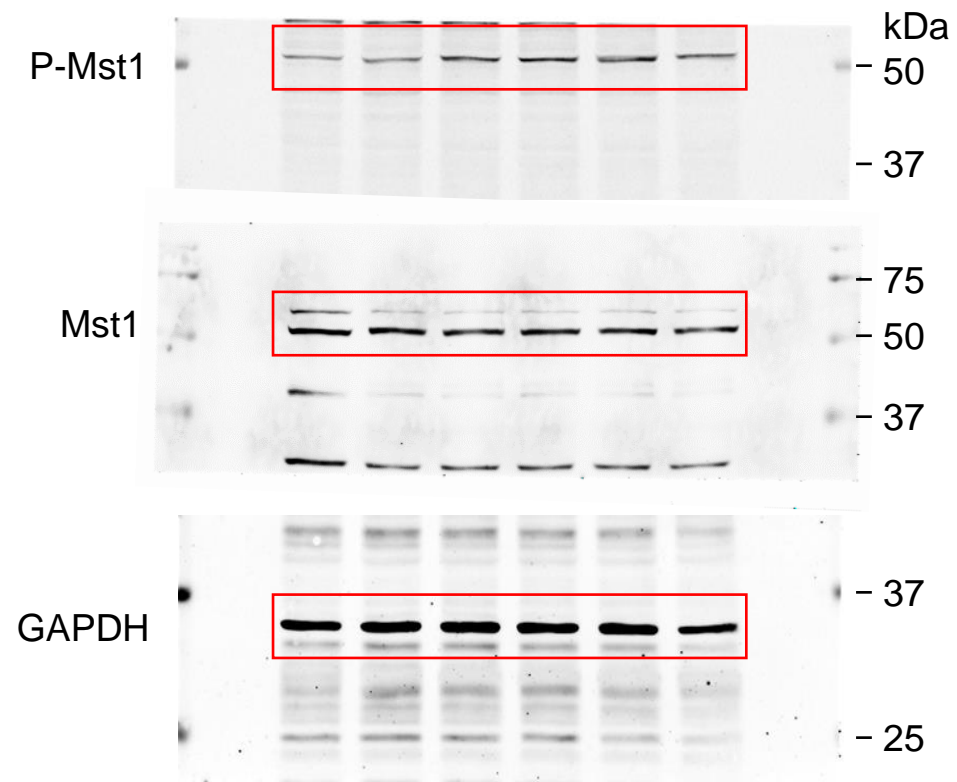

Fig. 1B

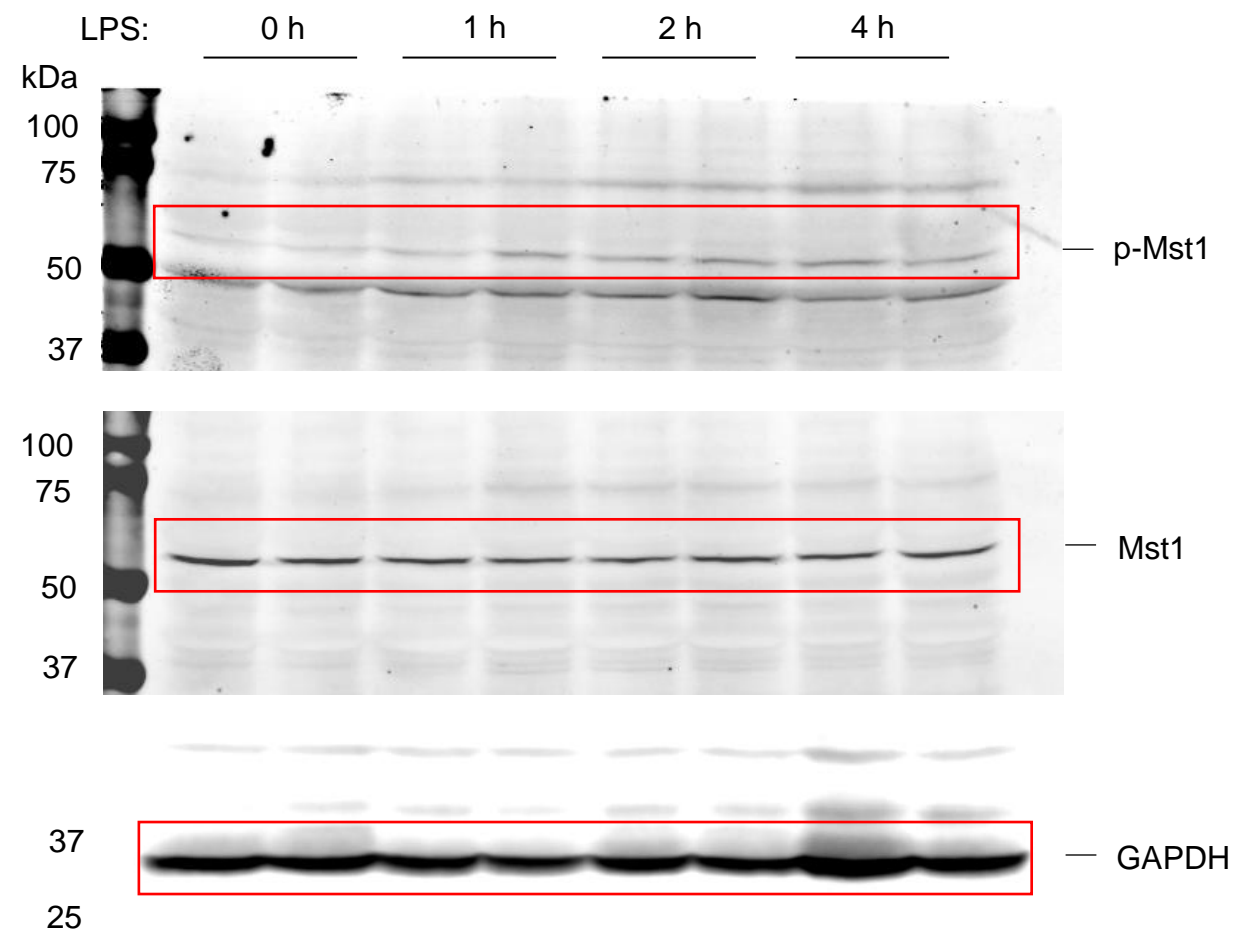

Fig.2E

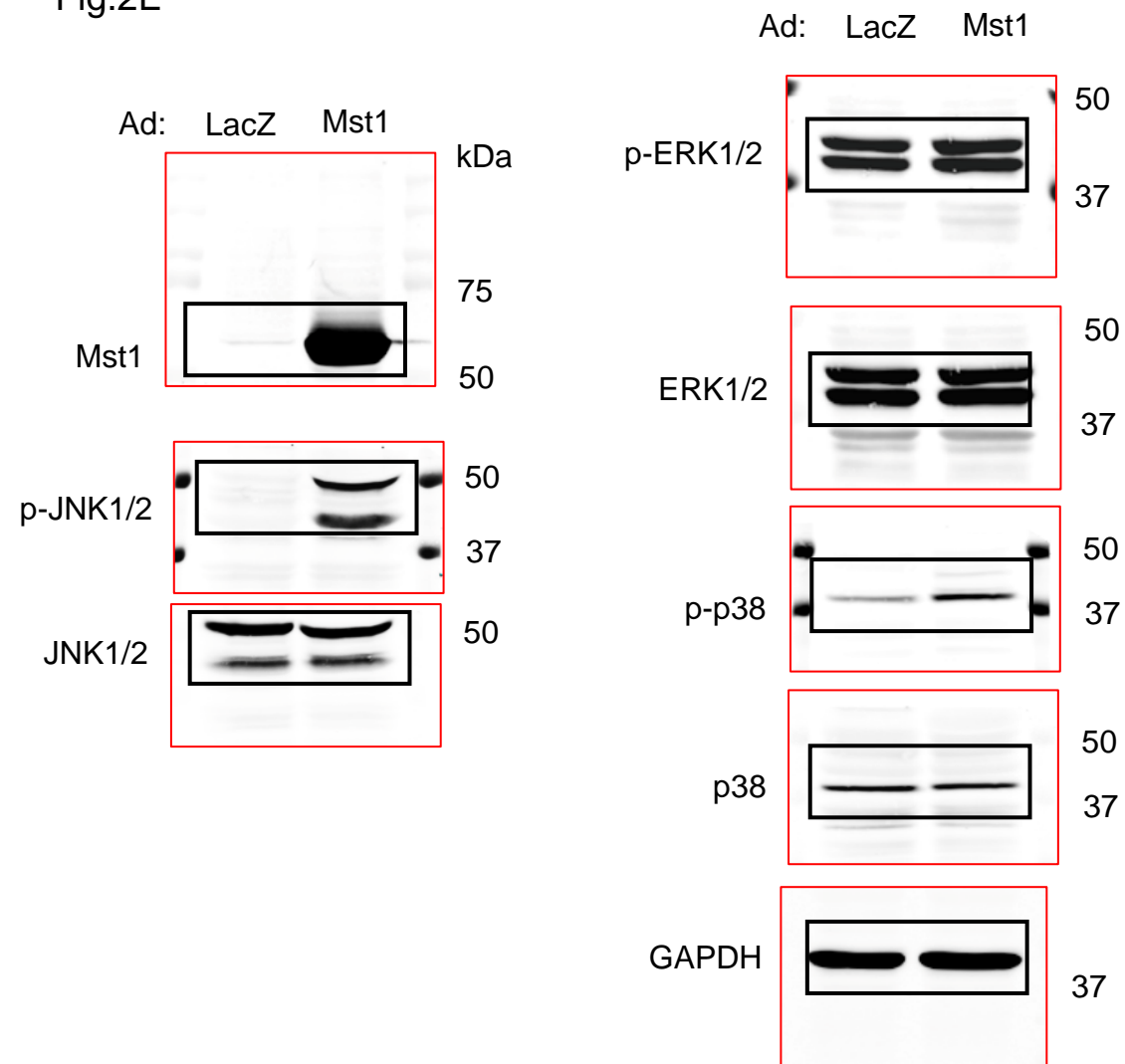

Fig. 3B

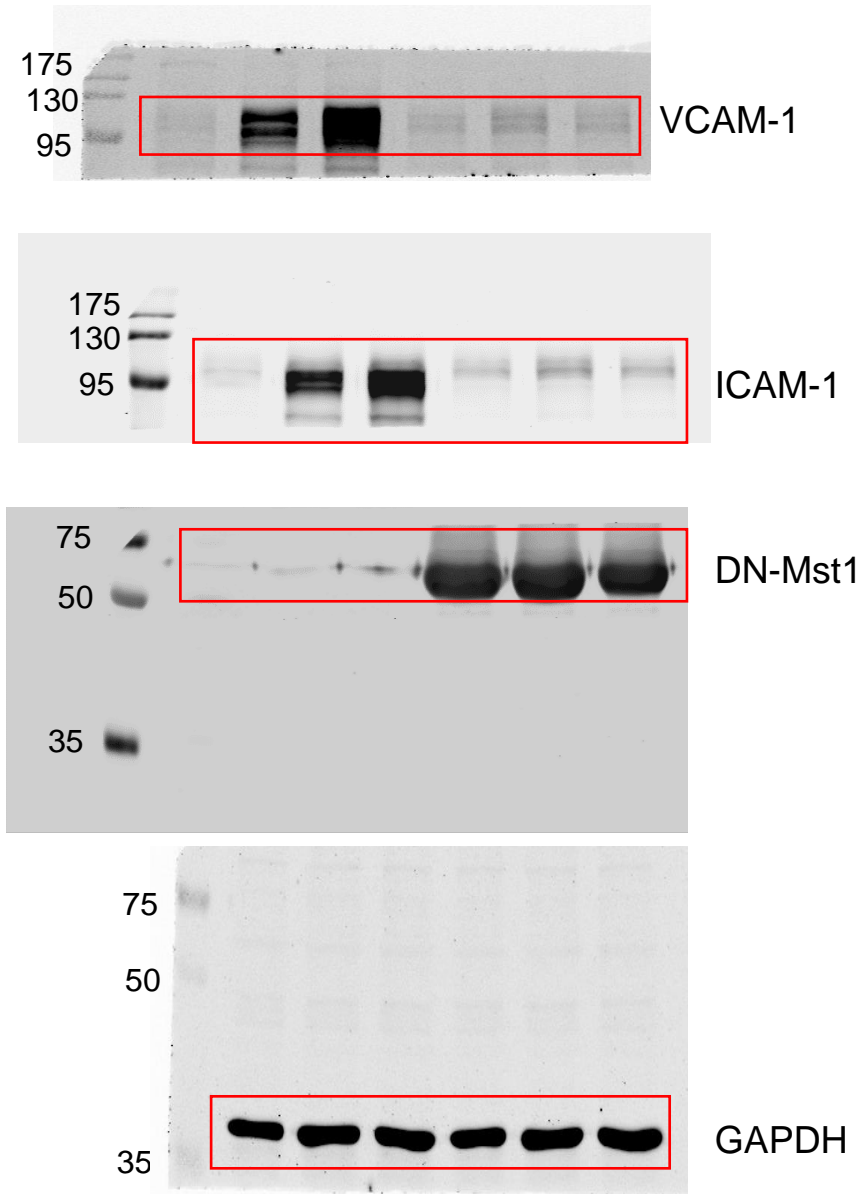

Fig. 4C

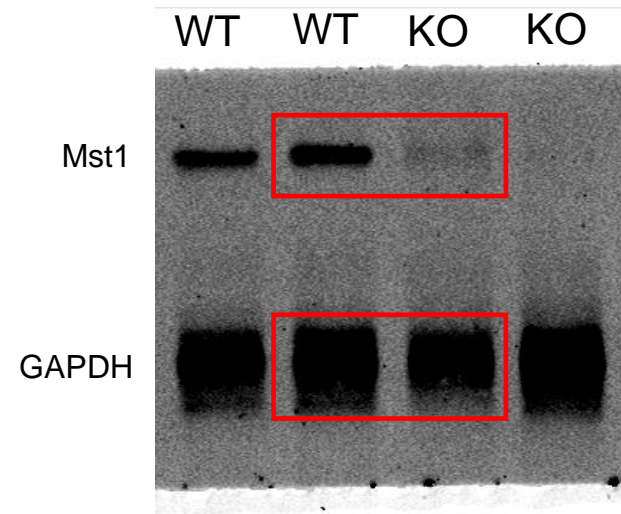

Fig. 5B

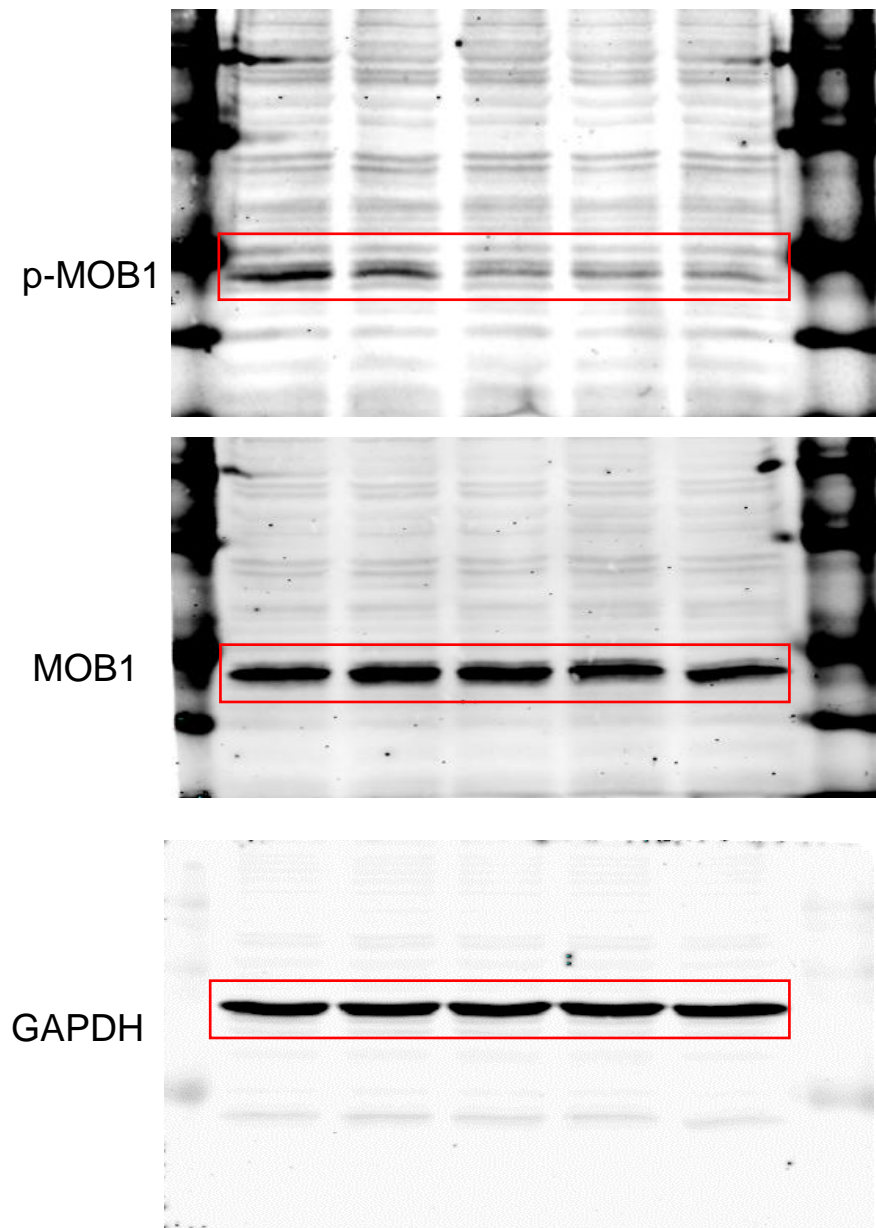

Fig. 5D

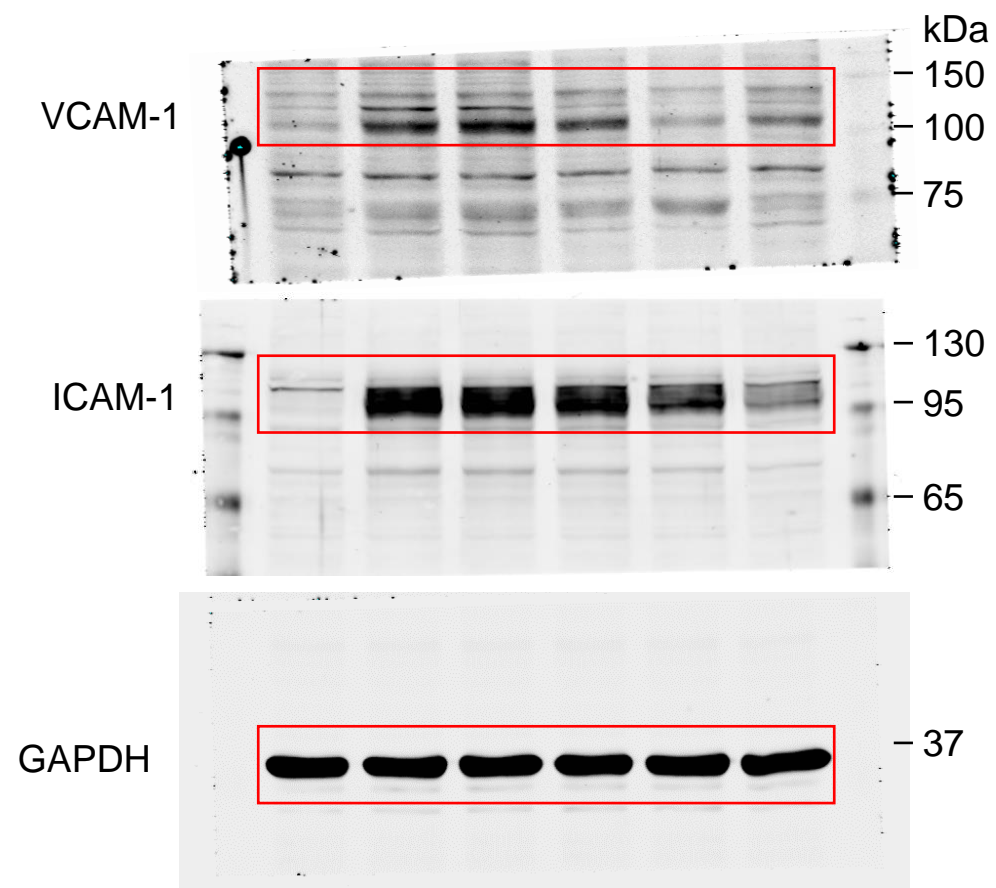

Fig. S1

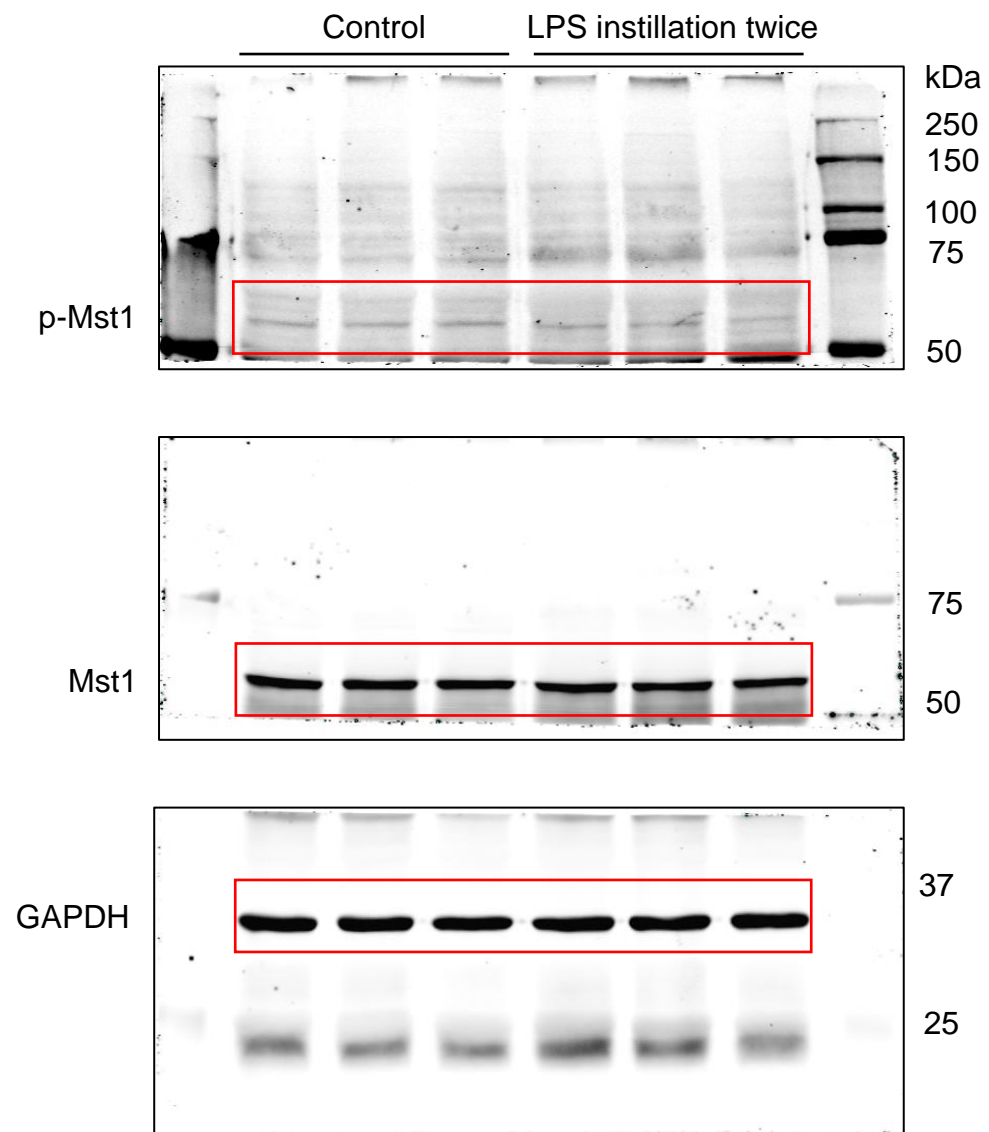

Supplement: Unedited blot and gel images [file jciinsight-9-178208-s175.pdf]
